# Variance reduction with synaptic density imaging in Parkinson’s disease using direct-4D PET image reconstruction

**DOI:** 10.1088/1361-6560/ae520a

**Published:** 2026-03-25

**Authors:** Paul Gravel, Jean-Dominique Gallezot, Kathryn Fontaine, David Matuskey, Richard E Carson

**Affiliations:** Department of Radiology and Biomedical Imaging, Yale University, New Haven, CT, United States of America

**Keywords:** positron emission tomography, direct PET image reconstruction, kinetic modeling, PMOLAR-1T, [&lt, sup&gt, 11&lt, /sup&gt, C]UCB-J

## Abstract

*Objective.* Direct reconstruction (DR) of parametric images from dynamic positron emission tomography data has been shown to provide substantial noise reduction compared to the conventional indirect reconstruction (IR) approach where frames are first reconstructed and then voxel time-activity curves are fitted to a kinetic model. The main goal was to compare DR and IR, on both *within-subject* and *between-subject* variability. *Approach.* This work evaluated the Parametric motion-compensation OSEM List-mode algorithm for resolution-recovery-1T DR method, using multiple scans of Parkinson’s disease patients with [^11^C]UCB-J, a radioligand for synaptic vesicle glycoprotein 2A (SV2A), a marker for synaptic density. This was achieved by comparing *K*_1_, *k*_2_, and *V*_T_ parametric images estimated, at full- and lower-count levels (20%, 10%, and 5%), between DR and IR. *Main Results.* DR delivered considerable improvement, compared to IR, by substantially reducing variability for both *within-subject* and *between-subject* analyses, and dramatically reducing noise-induced bias for *K*_1_ and *V*_T_. Conversely, IR increased the *within-subject* variability for *K*_1_ by 79%–353% and for *V*_T_ by 62%–79% across the lower count levels (averaged over regions at matched iterations). The *between-subject* variability was also increased with IR over DR with an increase of 20%–221% for *K*_1_ and 45%–48% for *V*_T_. Even at the full-count level, the *between-subject* variability was slightly increased for *K*_1_ by 4%, but by 24% for *V*_T_. Furthermore, at 5% count level, DR delivered comparable variability to IR at 20% counts. The noise-induced %bias, relative to the full-count level, for IR was 3%–28% (from 20% to 5% count levels respectively) for *K*_1_ and 12%–31% for *V*_T_, whilst for DR the %bias was only 1% for *K*_1_ across count levels, and 7%–18% for *V*_T_. *Significance.* To the best of our knowledge, this is the first demonstration that direct-4D reconstruction delivers lower variability and bias not only for *within-subject* analysis, but also for *between-subject* analysis.

## 1. Introduction

Dynamic positron emission tomography (PET) imaging allows for the investigation of the distribution of a radiotracer in the body spatially and over time. Conventionally, PET data are reconstructed as a set of 3D images, framed as a function of time, to obtain the spatiotemporal distribution of a radiotracer in specific regions or voxels in the human body, e.g., within the brain. The time-activity curves (TACs) created from these framed images are then analyzed using tracer kinetic modeling techniques, to produce images of biological interest, such as glucose metabolic rates or binding potentials, which find applications in areas such as oncology or neuropsychiatry (Gallezot *et al* 2020).

However, each 3D image is reconstructed separately without considering the temporal relationship between each frame. At the voxel level or for small regions, this often delivers noisy TACs, which subsequently produce noisy parametric images. Different methods have been employed for denoising voxel-wise TACs and thus producing less noisy parametric images (Shidahara *et al* 2007, Floberg *et al* 2012, Lu *et al* 2012, Mohyud-Din *et al* 2015). More recently, with the advent of artificial intelligence in medical imaging, deep-learning methods have gained in popularity for such denoising purposes (Li *et al* 2023, Reader and Pan 2023, Liu *et al* 2024).

Nevertheless, techniques which include kinetic models to directly reconstruct parametric images within a 4D image reconstruction framework have also been of interest (Snyder 1984, Carson and Lange 1985, Matthews *et al* 1997, Kamasak *et al* 2005, Reader and Verhaeghe 2014). These methods provide better signal-to-noise ratio due to, not only taking into account the temporal relationship across the full scan length, but also likely due to the more accurate modeling of the Poisson nature of the raw PET data.

Several studies have demonstrated the benefits of variance reduction offered by direct-4D PET image reconstruction over conventional indirect-3D techniques (Tsoumpas *et al* 2008, Rahmim *et al* 2009, Wang and Qi 2009, 2012, Gravel and Reader 2015, Petibon *et al* 2020, Marin *et al* 2025). However, most of these studies focused on simulations or within-subject metrics using a set of noisy realizations derived from one reference image. One such direct parametric image reconstruction method has been developed by our group which makes use of the 1-tissue compartment tracer kinetic model (Yan *et al* 2012), and was further extended to include event-by-event motion correction (Germino *et al* 2017). The goal of this work is to apply this direct method and assess its impact, in comparison to indirect-3D reconstruction, on multiple subjects. Here we evaluate both within-subject and between-subject variability for multiple real Parkinson’s disease (PD) patients scanned with [^11^C]UCB-J, a radioligand for synaptic vesicle glycoprotein 2A (SV2A), a marker for synaptic density (Finnema *et al* 2016). We also assess the effects of low-dose studies (by downsampling the list mode files) and the effect of iteration number. In addition, we investigate bias and variability not only at the voxel level, but also at the regions-of-interest (ROI) level, where ROI values are extracted from the parametric image. To our knowledge, this is the first study to compare the performance of DR with indirect, not only for *within-subject* variability, i.e., across noisy realizations, but also for *between-subject*, in addition to extending the analysis from voxel-level to ROI-level. We hypothesized that DR would reduce both kinds of variability, especially in low-dose scenarios.

## 2. Material and methods

### 2.1. PET scanning protocol

For this work, eight subjects (4F/4M, 52–74 y) diagnosed with mild to moderate PD (Hoehn-Yahr Stage: 2.0 ± 0.0, averaged total UPDRS Score 56 ± 15; mild bilateral disease) underwent PET imaging with [^11^C]UCB-J, as previously published (Matuskey *et al* 2020). Each subject was scanned for at least 60 min after a bolus injection on the high resolution research tomograph (HRRT) (Siemens/CTI, Knoxville, TN, USA) (de Jong *et al* 2007), preceded by a 6 min transmission scan for attenuation correction and scatter estimation. The mean injected activity was 596 ± 172 MBq and mean injected mass was 0.023 ± 0.010 *µ*g kg^−1^. During the PET sessions, continuous head motion data were acquired using the Polaris Vicra optical tracking system (NDI Systems, Waterloo, Canada). Arterial blood was collected for measurement of blood and plasma radioactivity. Corrections were made for metabolites as previously described (Finnema *et al* 2018).

The data in this work were obtained from a study performed under a protocol approved by the Yale University Human Investigation Committee and the Yale University Radiation Safety Committee, and in accordance with the US federal policy for the protection of human research subjects contained in title 45 part 46 of the code of federal regulations. Written informed consent was obtained from all participants after complete explanation of study procedures (Matuskey *et al* 2020).

### 2.2. One-tissue compartment (1TC) model

The 1TC model best describes the kinetic behavior of [^11^C]UCB-J (Finnema *et al* 2018) and is given by the following operational equation (Gunn *et al* 2015):

\begin{equation*}{C_{\mathrm{T}}}\left( t \right) = {K_1}{\mathrm{exp}}\left( { - {k_2}t} \right) \otimes {C_{\mathrm{p}}}\left( t \right)\end{equation*}

where:

*C*_T_(*t*) is the concentration of the radioligand in tissue (Bq ml^−1^) as a function of time, i.e. the tissue TAC;

*C*_P_(*t*) is the metabolite-corrected concentration of the radioligand in arterial plasma (Bq ml^−1^), i.e. the input function;

*K*_1_ is the rate constant at which the radioligand moves from the arterial plasma to tissue (ml min^−1^ cm^−3^);

*k*_2_ is the rate constant at which the radioligand moves from the tissue back to the blood circulation (min^−1^).

Equation (1) can be used to estimate *K*_1_ and *k*_2_ using weighted least-squares after reconstruction, as performed for conventional indirect reconstruction (IR). Alternatively, estimates of *K*_1_ and *k*_2_ can be estimated during reconstruction within the EM framework as done for DR (Germino *et al* 2017).

For this work, parametric images of *K*_1_ (ml min^−1^ cm^−3^) and *k*_2_ (min^−1^) were estimated using the 1TC model with a basis function implementation (unless otherwise specified) (Lodge *et al* 2000, Yan *et al* 2012, Germino *et al* 2017) with 500 bases using a *k*_2_ range logarithmically spaced between 0.005 and 1.0 min^−1^. For the IR, weights were based on the noise-equivalent counts of each frame. Parametric images of *V*_T_ (ml cm^−3^), the equilibrium ratio of tissue to plasma activity, were subsequently calculated as the ratio of *K*_1_/*k*_2_. *K*_1_ and *V*_T_ are the more important physiological parameters, as they relate to perfusion and synaptic density, respectively.

### 2.3. PET image reconstruction

IR (referred to hereafter as IR) was performed with an ordered-subset expectation-maximization algorithm (OSEM) using the motion-compensation OSEM List-mode algorithm for resolution-recovery (MOLAR) (Johnson *et al* 2004) reconstruction software with frame definition of 6 × 30 s, 3 × 1 min, 2 × 2 min, 10 × 5 min for a total of 21 frames over 60 min, followed by voxel-wise estimation of *K*_1_ and *V*_T_ using the 1TC model, skipping the first 2 frames to minimize effect of tracer delivery and blood volume contributions. DR (referred to hereafter as DR) was performed with Parametric-MOLAR software for the 1TC model (PMOLAR-1T) (Yan *et al* 2012, Germino *et al* 2017), which is frameless and directly delivers parametric images of *K*_1_ and *k*_2_, from which *V*_T_ (=*K*_1_/*k*_2_) is calculated. Both reconstruction methods were performed with 30 subsets and up to 4 iterations, including corrections for attenuation, normalization, scatter and randoms events, dead time, resolution modeling (spatially invariant) of the point spread function (PSF) due to resolution degradation effects (e.g., detector size) and event-by-event motion correction (Jin *et al* 2013). The same motion correction method and measured motion parameters were applied for both IR and DR Randoms contributions were estimated based on time-varying block singles rates, as opposed to delayed events. Scatter was estimated based on the single-scatter simulation (SSS) method (Watson 2000) across coarsely sampled detector space, and updated iteratively at subsets 0, 4, and 10 of the first iteration. For each time point, global scatter scale factors were determined as the ratio between the scatter measured just outside the attenuating material and the corresponding scatter estimate. For DR, SSS scatter was estimated at 19 time points corresponding to the mid-frame times of the IR method (skipping first two IR frames), using an emission image created for each time point, by using the *K*_1_ and *k*_2_ parameters estimated within the DR framework at the current subset, and ${C_{\mathrm{p}}}\left( t \right)$. Finally, linear interpolation among the 19 estimated time points was used to determine the scatter estimate for each event (Yan *et al* 2012, Germino *et al* 2017).

In addition, each subject’s list-mode file was downsampled by 20%, 10%, and 5% of the total counts, to create a set of 5, 10 and 10 independent noisy replicates (as used previously (Gravel and Reader 2015, Germino *et al* 2017)) respectively, to evaluate the impact of DR on bias and variability across those noisy replicates in comparison to conventional IR. This downsampling was achieved by applying a repeating sequence of 50 ms gates for the 60 min scan duration, with each 5th, 10th, or 20th gate kept per replicate, for 20%, 10%, and 5% downsampling, respectively.

### 2.4. magnetic resonance image (MRI) acquisition

For definition of ROIs and PET image co-registration, each subject received a structural MRI acquired on a 3T Prisma system (Siemens Medical Systems, Erlangen, Germany) using a T1-weighted magnetization-prepared rapid gradient echo sequence with a 3D-isotropic resolution of 1 mm^3^ (flip angle = 7°, echo time = 3.34 ms, inversion time = 1100 ms, repetition time = 2500 ms).

### 2.5. Image co-registration and ROI delineation

Each parametric image was resampled from native space (256 × 256 × 207 voxels of 1.22 × 1.22 × 1.23 mm^3^) to stereotaxic space (MNI space: 91 × 109 × 91 voxels of 2 × 2 × 2 mm^3^) (Collins *et al* 1998) using tri-linear interpolation and a transformation obtained from the concatenation of a 6-parameter linear transformation matrix estimated with a mutual information objective function between the summed PET image (0–10 min) and MRI (using FLIRT/FSL), and a non-linear transformation matrix estimated between the subject MRI and a template MRI in MNI space (using bioimage suite software v3.01). A total of 8 ROIs were delineated: 7 in gray matter (GM) and 1 in white matter (WM). ROIs for: frontal cortex, occipital cortex, caudate nucleus, putamen, hippocampus, and cerebellum, were extracted from the automatic anatomical labeling (AAL) template (Tzourio-Mazoyer *et al* 2002). The substantia nigra, a primary target region in PD, was defined from a previous study using [^11^C-]-(+)-PHNO (a dopamine D_2_/D_3_ agonist radioligand) scans (Gallezot *et al* 2014). The centrum semiovale, a WM reference region for SV2A-PET, was defined as described in Rossano *et al* (2019).

### 2.6. Figures of merit

To assess the performance between IR and DR, metrics at the voxel-level as well as at the ROI-level were defined and applied for each reconstruction method. These were calculated in two ways: ***within-subject***, i.e. across noisy realizations for each subject, and ***between-subject***, i.e. across subjects for each noisy realization. Between-subject values were also determined for the full count data set. For consistency, all results were extracted in MNI space, unless otherwise specified.

#### 2.6.1. Voxel-wise metrics

##### 2.6.1.1. Within-subject metrics

At the voxel-level, the mean ($m$) and standard-deviation ($\sigma $) were calculated for each voxel (*j*) contained in each parametric $\theta {\text{ }}\left( { = {V_{\mathrm{T}}}{\text{ or }}{K_1}} \right)$ map, across noisy realizations (*r*), at each iteration, for each subject ($s$), i.e. ***within-subject***, respectively using:

\begin{equation*}{m_{js}} = \mathop \sum \limits_{r = 1}^{{N_{\mathrm{R}}}} \frac{{{\theta _{jrs}}}}{{{N_{\mathrm{R}}}}};\end{equation*}

\begin{equation*}{\sigma _{js}} = \sqrt {\frac{{\mathop \sum \nolimits_{r = 1}^{{N_{\mathrm{R}}}} {{\left( {{\theta _{jrs}} - {m_{js}}} \right)}^2}}}{{{N_{\mathrm{R}}} - 1}}} \end{equation*}

where ${N_{\mathrm{R}}}$ is the number of noisy realizations.

These metrics were then averaged across subjects using:

\begin{equation*}{m_j} = \mathop \sum \limits_{s = 1}^{{N_{\mathrm{S}}}} \frac{{{m_{js}}}}{{{N_{\mathrm{S}}}}};\end{equation*}

\begin{equation*}{\sigma _j} = \sqrt {\mathop \sum \limits_{s = 1}^{{N_{\mathrm{S}}}} \frac{{\sigma _{js}^2}}{{{N_{\mathrm{S}}}}}} \end{equation*}

where ${N_{\mathrm{S}}}$ is the number of subjects. Here, ${\sigma _j}$ reflects the subject-average standard deviation of voxel *j* across realizations.

##### 2.6.1.2. Between-subject metrics

The performance of the reconstruction methods was also assessed ***between-subject*** using a change of variables in equations (4) and (5), for which the subscripts *s* and *r* are interchanged. The equations become:

\begin{equation*}{\tilde m_{jr}} = \mathop \sum \limits_{s = 1}^{{N_{\mathrm{S}}}} \frac{{{\theta _{jrs}}}}{{{N_{\mathrm{S}}}}};\end{equation*}

\begin{equation*}{\tilde \sigma _{jr}} = \sqrt {\frac{{\mathop \sum \nolimits_{s = 1}^{{N_{\mathrm{S}}}} {{\left( {{\theta _{jrs}} - {{\tilde m}_{jr}}} \right)}^2}}}{{{N_{\mathrm{S}}} - 1}}} \end{equation*}

where $\tilde m$ and $\tilde \sigma $ are now calculated across (or between-) subjects, for each realization, and then averaged across realizations using:

\begin{equation*}{\tilde m_j} = \mathop \sum \limits_{r = 1}^{{N_{\mathrm{R}}}} \frac{{{{\tilde m}_{jr}}}}{{{N_{\mathrm{R}}}}};\end{equation*}

\begin{equation*}{\tilde \sigma _j} = \sqrt {\mathop \sum \limits_{r = 1}^{{N_{\mathrm{R}}}} \frac{{\tilde \sigma _{jr}^2}}{{{N_{\mathrm{R}}}}}} .\end{equation*}

Here, ${\tilde \sigma _j}$ reflects the replicate-average standard deviation of voxel *j* across subjects.

Since the mean is a linear operation, and because all images are in MNI space, the within-subject mean and between-subject mean will be the same, i.e., ${m_j} = {\tilde m_j}$. Finally, to quantify the regional voxel-wise performance, each of the above metrics was averaged across voxels in each ROI (denoted by $\langle \rangle $, i.e., ${m_{{\mathrm{ROI}}}} = {\langle {m_j}\rangle _{j \in {\mathrm{ROI}}}}$, ${\sigma _{{\mathrm{ROI}}}} = {\langle {\sigma _j}\rangle _{j \in {\mathrm{ROI}}}}$, ${\tilde m_{\mathrm{ROI}}} = {\tilde m_{j \in {\mathrm{ROI}}}}$, ${\tilde \sigma _{\mathrm{ROI}}} = {\tilde \sigma _{j \in {\mathrm{ROI}}}}$, and the variability is reported as the percent coefficient of variation (%CV), i.e., within-subject $\% C{V_{{\mathrm{ROI}}}} = 100 \times {\sigma _{{\mathrm{ROI}}}}/{m_{{\mathrm{ROI}}}}$, and between-subject $\% {\mathop {CV}\limits^\sim _{\mathrm{ROI}}} = 100 \times {\tilde \sigma _{\mathrm{ROI}}}/{\tilde m_{\mathrm{ROI}}}$, for which ${m_{{\mathrm{ROI}}}}$ =${\tilde m_{\mathrm{ROI}}}$ was set to the corresponding ROI value obtained with the conventional IR method at full counts at iteration 2, which is the iteration at which our HRRT results are conventionally reported.

##### 2.6.1.3. TAC simulation

Nonlinear modeling applied to noisy data can introduce bias in the parameter estimates. Therefore, a TAC simulation was performed to investigate noise-induced bias as a function of iteration and noise level for comparison to the IR results. This simulation consisted of creating a TAC for each ROI (equation (1)), using the mean *K*_1_ and *V*_T_ ROI values (with *k*_2_ = *K*_1_/*V*_T_) from the full count IR parametric images at iteration 2 (as used in practice (Holmes *et al* 2024)). For each TAC, 10 000 realizations with Gaussian noise were simulated. The noise level was calculated using the voxel SD across noisy replicates per frame derived from the real data of a representative subject; these SD values were averaged over the voxels of each region. This calculation was repeated for each of the count levels at each iteration. For the full count level, since no replicates are available, the SD values for the 20% and 10% count levels per frame, were divided by sqrt(5) and sqrt(10), respectively, and then averaged. Each noisy TAC was then fitted using the 1TC model (the same basis function implementation used for the human data) to estimate *K*_1_ and *k*_2_ (with *V*_T_ = *K*_1_/*k*_2_), using weights equal to 1/SD^2^, and then averaged across the 10 000 replicates.

This simulation provides some guidance for interpretation of IR parametric images which uses the same fitting algorithm on a voxel-wise level.

#### 2.6.2. ROI metrics

In addition to voxel-level metrics, voxels contained in each ROI were first averaged for each parametric map (*K*_1_, *V*_T_ or *k*_2_), and then the mean and SD (with %CV calculated as above) were calculated to assess both within-subject and between-subject variation. This differs from calculating the SD/%CV at the voxel-level and then averaging these values for each ROI as performed in the above section. Since the mean is a linear operation, the voxel-wise mean and ROI mean will be the same.

## 3. Results

Here we used up to 4 iterations (with 30 subsets) for both IR and DR; this choice was justified by the per-iteration changes in *K*_1_ and *k*_2_ values being less than 2% (averaged across GM ROIs) after 3 iterations at the full count level for both reconstruction methods (tables S1 and S2); similar results were observed in (Germino *et al* 2017). The corresponding changes in *V*_T_ were slightly higher: percent change between iteration 4 vs iteration 3 for IR = 4.0% ± 0.9%, and for DR = 2.8% ± 1.3% (table S3). In all cases, the per-iteration %changes for IR were greater than for DR

### 3.1. Average kinetic parameters

#### 3.1.1. Human data

**Figure 1.**
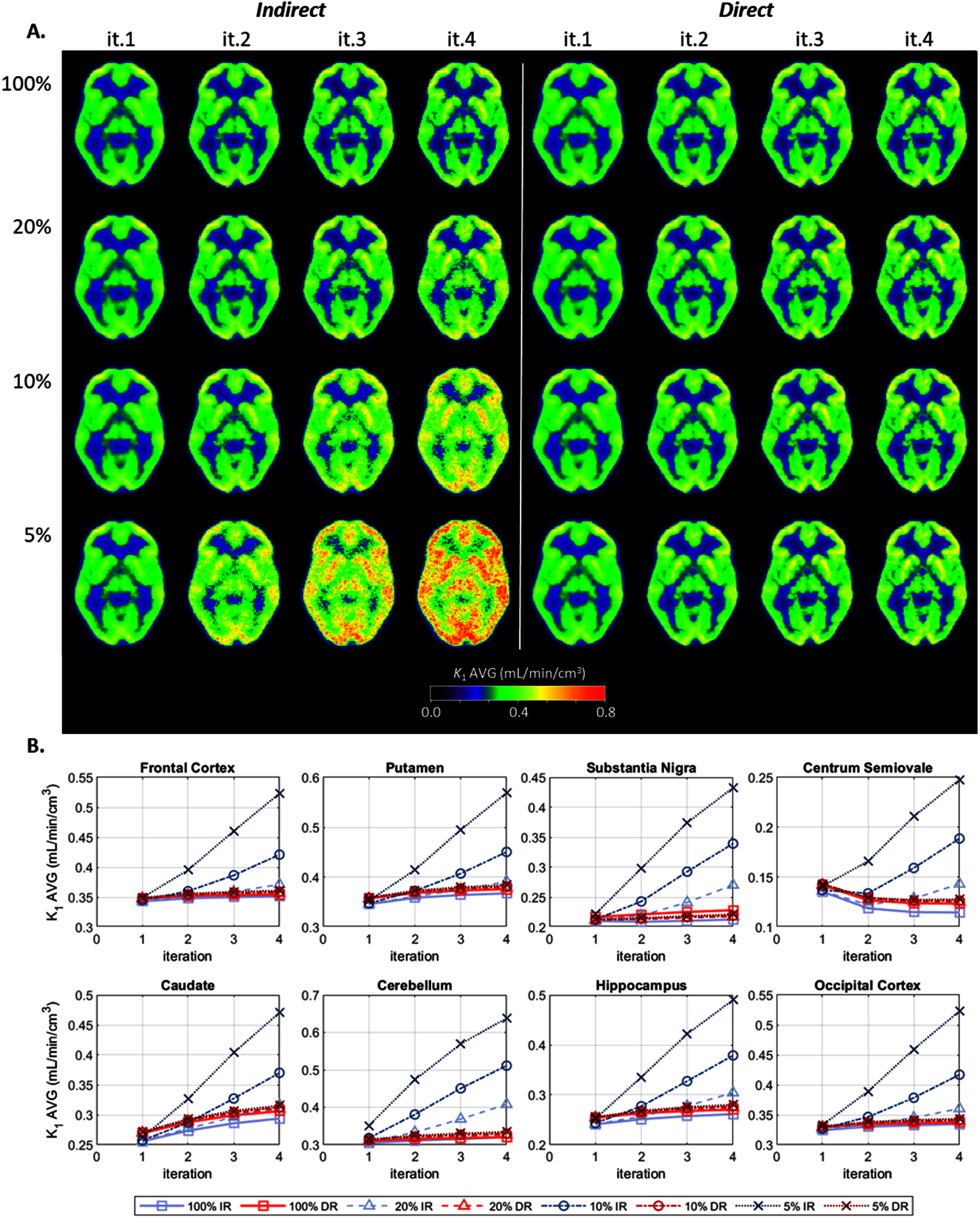
(A) *K*_1_ average images for IR (left) and DR (right) methods, for iterations 1–4, and for each count level: 100% (*n* = 1), 20% (*n* = 5), 10% (*n* = 10), and 5% (*n* = 10). (B) Average values for each ROI as a function of iteration. The *y*-axis range varies across plots.

Figure 1(A) displays the intersubject averaged *K*_1_ parametric images for each count level and iteration. Figure 1(B) shows the regional *K*_1_ changes, for the 8 ROIs, as a function of iteration, for each count level.

At the full count level, the images (figure 1(A)) are visually comparable between IR and DR This is confirmed in figure 1(B), for the GM ROIs, with the blue squares (IR) and red squares (DR) being close together. DR delivered slightly higher *K*_1_ values (AVG ± SD across 7 GM ROIs and 8 subjects): 3% ± 2% at iteration 2 and 3% ± 3% at iteration 4. The small increases observed in *K*_1_ with iteration for the GM ROIs were balanced by the decrease in the WM as shown in the centrum semiovale (Rossano *et al* 2019).

For DR, the image intensity is visually uniform across count levels and across iterations (figure 1(A)), denoted by the red lines (figure 1(B)), for the GM ROIs, overlapping across count levels and being almost flat across iterations. The %bias (relative to DR full counts at iteration 2) across count levels was small: 1% ± 2% at iteration 2 and 5% ± 3% at iteration 4.

For IR, on the other hand, there is an increase in *K*_1_ values as the image noise gets higher (figure 1(A)), either due to lower counts or higher iterations, denoted by the blue lines (figure 1(B)) diverging from the full counts. For the 20% count level (triangles), the %bias of GM values (relative to IR full counts at iteration 2) was 3% ± 2% at iteration 2 and 17% ± 10% at iteration 4. At the 5% count level (‘×’), the %bias increased to 28% ± 15% at iteration 2 and 78% ± 24% at iteration 4. This pattern is consistent with noise-induced bias (see section 3.1.2 Simulated TAC data).

Similar behavior was observed for *k*_2_, but with higher bias values (see supplement figure S1). Compared to GM, higher *k*_2_ values can be observed in WM. High *k*_2_ values on the border of the brain are found artifactually in voxels with low *K*_1_ and *V*_T_ values. Similar behavior can be observed for single-subject *k*_2_ images, as well as for *k*_2_ SD images (see below).

**Figure 2.**
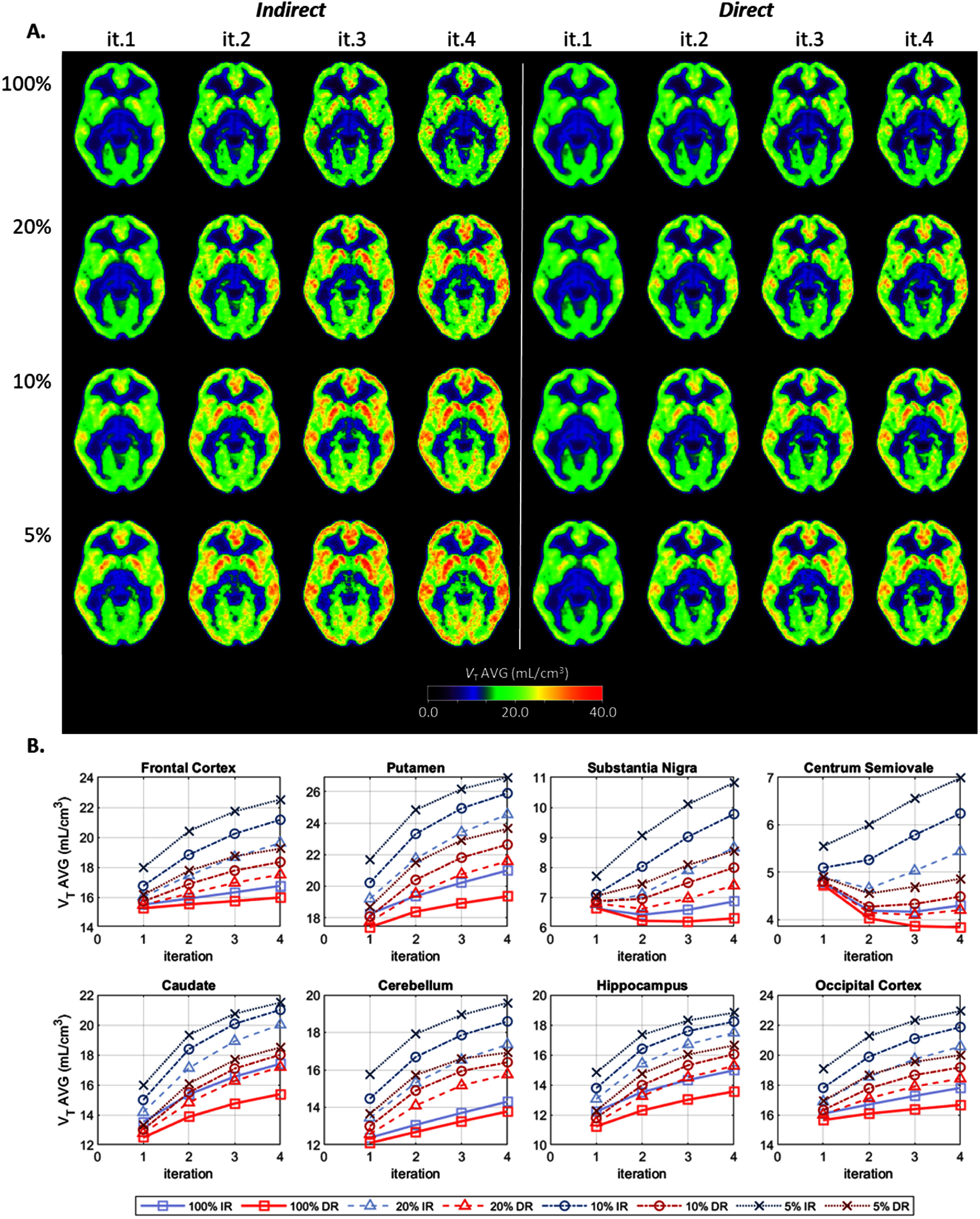
(A) *V*_T_ average images, for IR (left) and DR (right) methods, for iterations 1–4, and for each count level: 100% (*n* = 1), 20% (*n* = 5), 10% (*n* = 10), and 5% (*n* = 10). (B) Average values for each ROI as a function of iteration. The *y*-axis range varies across plots.

Figure 2(A) displays the intersubject averaged *V*_T_ parametric images for each count level and iteration. Figure 2(B) shows the regional *V*_T_ changes, for the 8 ROIs, as a function of iteration, for each count level.

At the full count level, the images for DR (figure 2(A)) have slightly lower intensity compared to those for IR. This is confirmed in figure 2(B) with the red squares (DR) being below the blue squares (IR). The percent difference (DR relative to IR) across GM ROIs was −5% ± 3% at iteration 2 and −7% ± 3% at iteration 4 as previously observed (Germino *et al* 2017). The small increases observed in *V*_T_ with iteration for the GM ROIs is balanced by the decrease in the WM as shown in the centrum semiovale (Rossano *et al* 2019), as observed for *K*_1_.

For the lower count levels, the intensity of the images (figure 2(A)) increases with noise levels for both DR and IR methods, but with IR exhibiting a larger effect. At 20%count iteration 2, the %bias was 12% ± 3% for IR and 7 ± 2% for DR, and at 5% counts the %bias was 31% ± 6% for IR and 18% ± 3% for DR, for the GM ROIs. At 20% count iteration 4, this %bias increased to 29 ± 5% for IR and 19 ± 5% for DR, and at 5% counts it increased to 45% ± 11% for IR and 31% ± 5% for DR

For the centrum semiovale, the observed bias was also less pronounced for DR with an increase of 3% (20% counts) and 13% (5% counts) at iteration 2. Correspondingly for IR at iteration 2, this increase was 11% (20% counts) and 43% (5% counts).

Supplement figures S2–S4 show corresponding parametric images (*K*_1_, *k*_2_, and *V*_T_, respectively) for a single subject. When data from individual subjects are plotted, the pattern of biases with iteration and injected dose were consistent with the intersubject averages shown in figure 1 (*K*_1_), S1 (*k*_2_) and 2 (*V*_T_).

#### 3.1.2. Simulated TAC data

The goal of the simulation was to assess the effects of noise-induced bias, by simulating noisy data consistent with the noise level at each iteration and count level. Many aspects of the count level and iteration effects for IR in human data were replicated in the simulation. The regional changes for the average kinetic parameters as a function of iteration, for each count level, are shown in supplement figures S5 (*K*_1_), S6 (*k*_2_), and S7 (*V*_T_), with the plots for the real data shown in panel A (top) and the corresponding plots for the simulation in panel B (bottom). Note that the *x*-axis label of iteration and count level notations for the simulation data in these figures reflect that each simulation used a noise level based on reconstructed images at the respective count level and iteration.

For the simulated data with noise equivalent to full count data, the change between iteration 2 and 4 was less than 2% for both *K*_1_ and *k*_2_, across GM ROIs. For the simulated *V*_T_, this change was about 6%. These changes were mirrored in the real data, i.e., between iteration 3 and 4, the change in simulated *V*_T_ was 3.0% ± 1.5% (real data: 4.0% ± 0.9%). This confirms our choice of not using more than 4 iterations (30 subsets), since noise-induced bias was already present by iteration 4, even at the full count level.

At lower counts levels, noise-induced bias became larger. Table 1 summarizes the %bias (relative to simulated/real full counts at iteration 2) comparing the values between the simulation and real data sets across GM ROIs, at iteration 2 and 4 for the lower count levels.

**Table 1.** %Bias (median across 7 GM ROIs), relative to full counts at iteration 2, for the indirect reconstruction method as a function of count level and iteration for the simulation and real data sets.

			*K*_1_ parameter			*k*_2_ parameter			*V*_T_ parameter	
Counts	Iteration		Simulation	Real data		Simulation	Real data		Simulation	Real data
20%	2		1%	1%		2%	5%		9%	11%
	4		3%	16%		8%	37%		26%	29%
10%	2		2%	5%		5%	20%		20%	20%
	4		8%	35%		25%	110%		39%	35%
5%	2		5%	19%		14%	74%		34%	28%
	4		26%	72%		105%	212%		49%	40%

For all three kinetic parameters, the pattern was very similar between the simulated and real data sets with the values increasing with lower counts and more iterations. Although the simulation delivered similar biases for *V*_T_, the biases were smaller for both *K*_1_ and *k*_2_ when compared to the real data sets. We attribute this difference in bias, at least in part, to the difference in noise characteristics between the simulated and the real data. The simulation assumed Gaussian noise and used fitting weights based exactly on the simulated noise level. Conversely, the image noise for the real data is not Gaussian-distributed and the weights were approximated based on noise-equivalent counts. See Discussion for details.

### 3.2. Within-subject variability

#### 3.2.1. Voxel-wise within-subject variability

**Figure 3.**
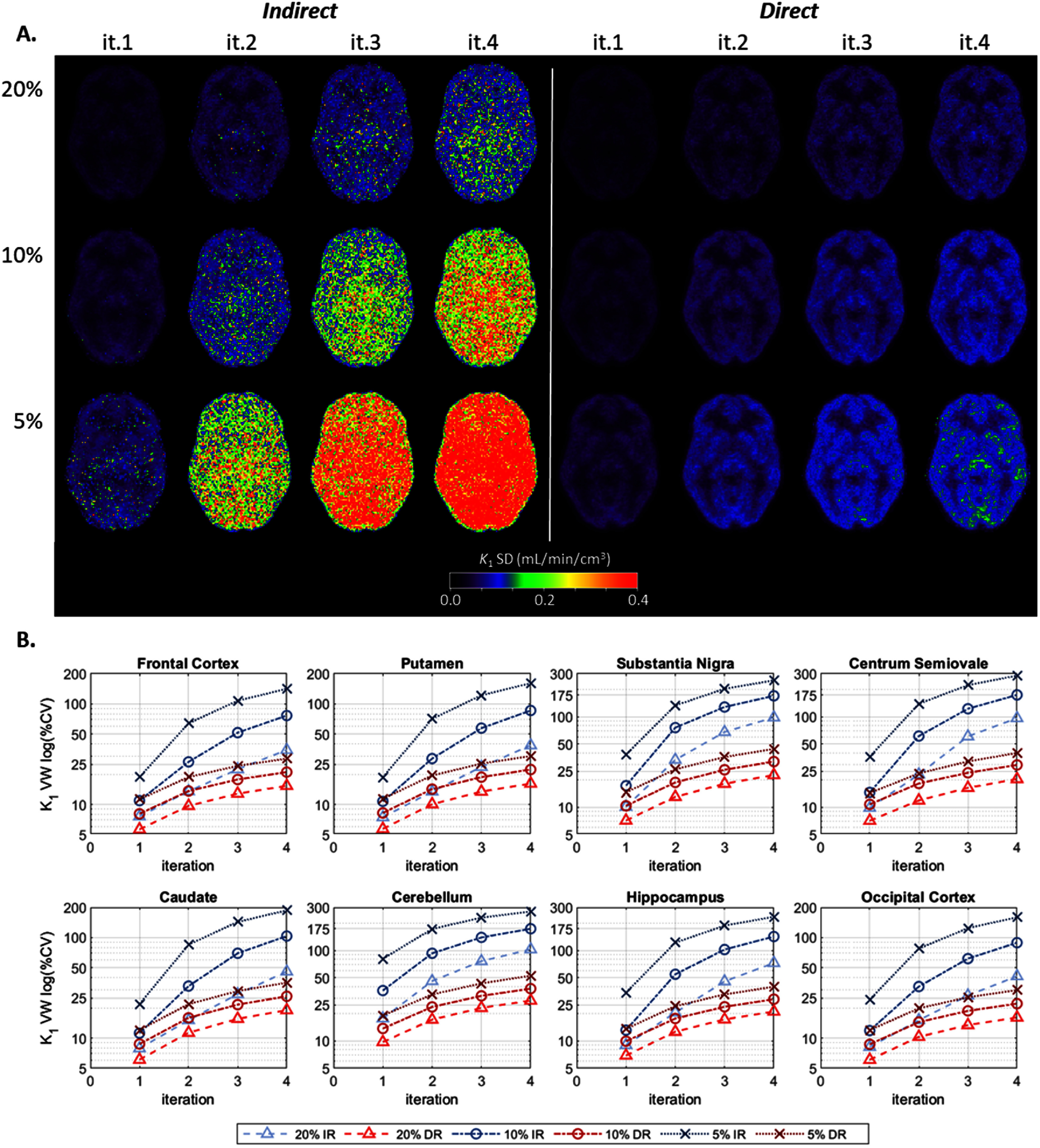
(A) *K*_1_ *within-subject* SD images calculated across the noisy replicates per count level (equations (2) and (3)) and then averaged across subjects (equations (4) and (5)), for IR (left) and DR (right) methods, for iterations 1–4, and for each lower count level (there are no SD images for the 100% count level since *n* = 1): 20% (*n* = 5), 10% (*n* = 10), and 5% (*n* = 10). (B) Voxel-wise (VW) percent coefficient of variation (%CV), calculated using corresponding ROI mean at iteration 2 of IR at full counts, for each ROI as a function of iteration. The *y*-axis is on a log-scale and its range varies across plots. Clearly the variability is lower for DR across all subjects.

Figure 3(A) displays the *within-subject* SD images, calculated across the noisy replicates per count level (equations (2) and (3)) and then averaged across subjects (equations (4) and (5)), for *K*_1_ per count level and each iteration. Figure 3(B) shows the corresponding voxel-wise (VW) %CV, for the 8 ROIs. The *K*_1_ variability is clearly higher for IR than DR At iteration 2, the average percent increase in variability delivered by IR (compared to DR) was 79V% ± 55% for the 20% count level and 353% ± 90% for the 5% count level. At iteration 4, this percent increase was 223% ± 95% and 460% ± 70% for the 20% and 5% counts respectively. The %CV delivered by DR at 5% counts, was comparable to, or even lower than, that of IR at 20% counts, i.e. DR provided a 4-fold count advantage likely due to modeling the inherent Poisson distribution of the data.

Similar behavior was observed for *k*_2_, but with higher %CV values (see supplement figure S8).

**Figure 4.**
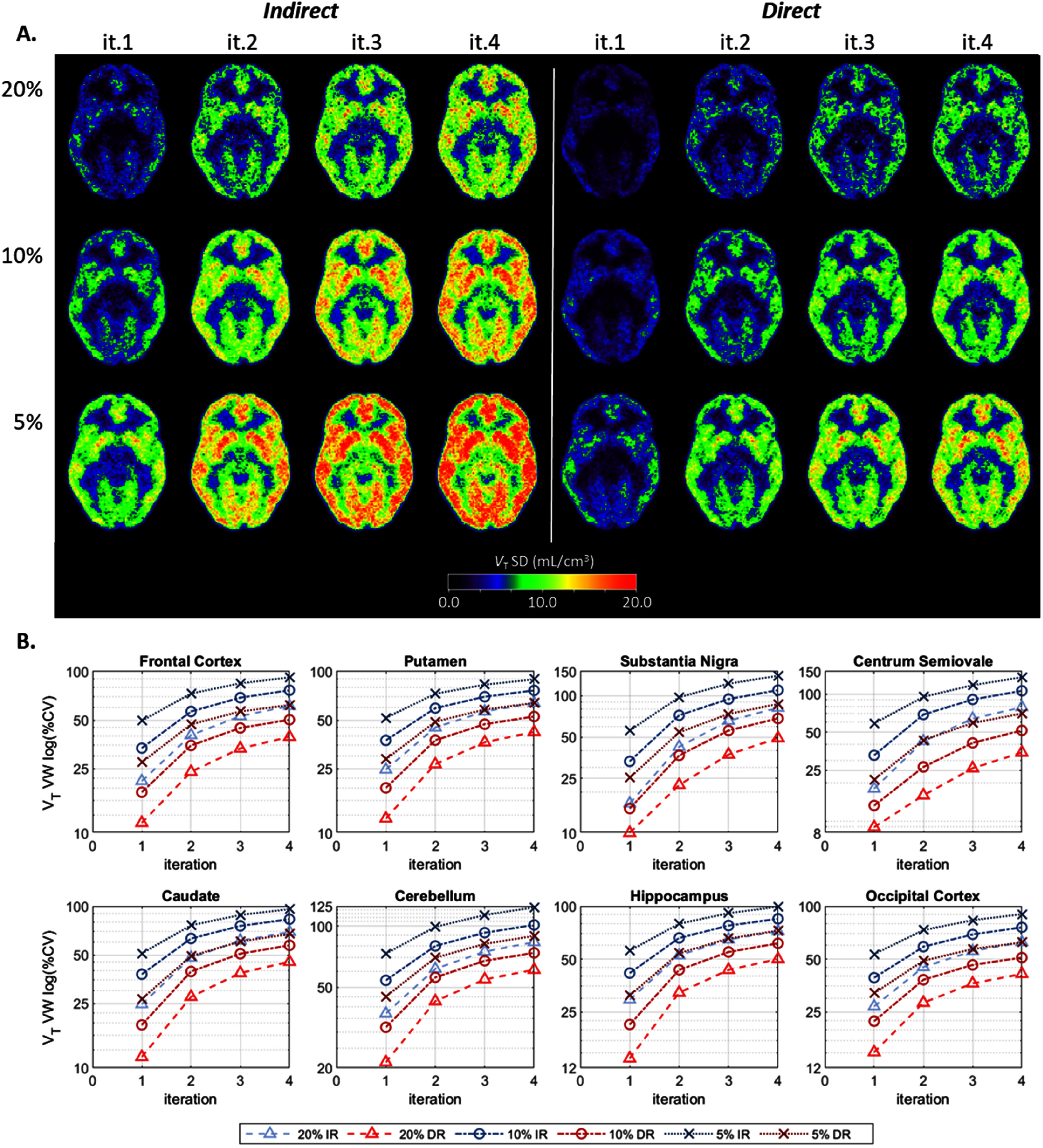
(A) *V*_T_ *within-subject* SD images, calculated across the noisy replicates per count level (equations (2) and (3)) and then averaged across subjects (equations (4) and (5)), for IR (left) and DR (right) methods, for iterations 1–4, and for each lower count level (there are no SD images for the 100% count level since *n* = 1): 20% (*n* = 5), 10% (*n* = 10), and 5% (*n* = 10). (B). Voxel-wise (VW) percent coefficient of variation (%CV), calculated using corresponding ROI mean at iteration 2 of IR at full counts, for each ROI as a function of iteration. The *y*-axis is on a log-scale and its range varies across plots. Clearly the variability is lower for DR across all subjects.

Figure 4(A) displays the *within-subject* SD parametric images for *V*_T_ per count level and each iteration. Figure 4(B) shows the corresponding voxel-wise (VW) %CV, for the 8 ROIs, as a function of iteration, for each count level. Although less pronounced than for *K*_1_ and *k*_2_, the *V*_T_ variability is still clearly higher for conventional IR compared to DR As observed for *K*_1_, the *V*_T_ %CV delivered by DR at 5% counts was also comparable to that of IR at 20% counts. At iteration 2, the percent increase in variability delivered by IR (compared to DR) was 79% ± 38% for the 20% count level and 62% ± 26% for the 5% count level, across all 8 ROIs. Interestingly, at iteration 4, this percent increase was smaller, 61% ± 28% and 51% ± 19% for the 20% and 5% counts respectively (see Discussion).

Ideally, for each kinetic parameter, the *within-subject* SD would increase by the square root of the relative decrease in counts, e.g. decreasing the counts from 20% to 10% should increase the SD by a factor of sqrt(2), and a decrease from 20% to 5% by a factor of 2. For *K*_1_ and *k*_2_, DR offered an increase in SD close to the expected factors. For *K*_1_, the increase in SD (averaged across ROIs and across iterations 2–4) was 1.40 (from 20% to 10%) and 1.91 (from 20% to 5%). Correspondingly for *k*_2_, these increases were 1.32 and 1.68. On the other hand, IR delivered an increase which was greater than the expected factor. For *K*_1_, the increase in SD was 2.15 (from 20% to 10%) and 4.25 (from 20% to 5%). Correspondingly for *k*_2_, these increases were 2.03 and 3.36. This again suggests that DR better handles noise in a manner more consistent with the Poisson distribution. For *V*_T_, the increase in SD was similar between both IR and DR, but with DR slightly closer to the expected factor, i.e. 1.31 for IR vs 1.37 for DR from 20% to 10%, and 1.62 for IR vs 1.76 for DR from 20% to 5%.

Figures S9–S11 show corresponding SD parametric images (*K*_1_, *V*_T_, and *k*_2_, respectively) for a single subject.

#### 3.2.2. ROI-level within-subject variability

**Figure 5.**
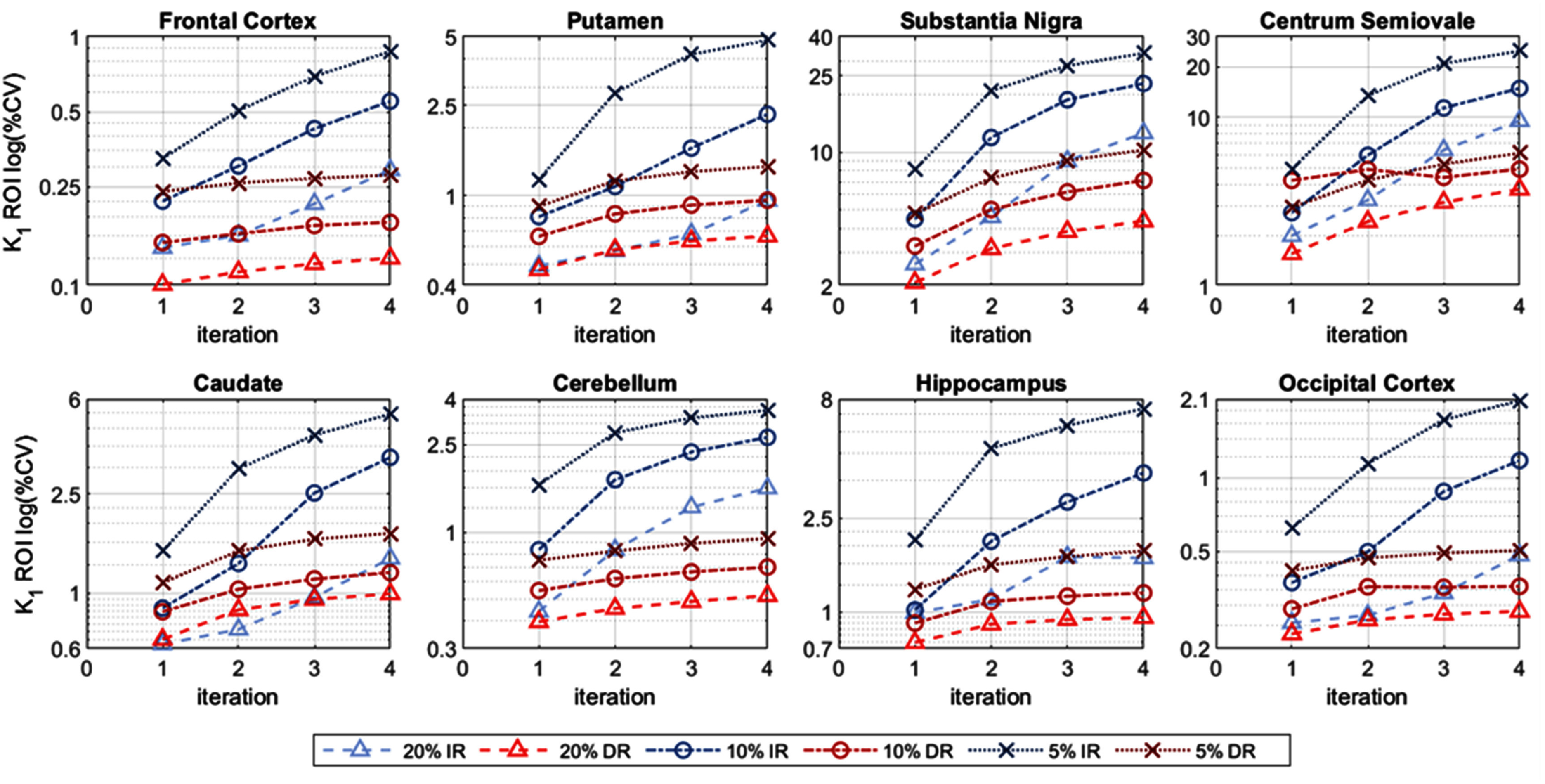
ROI-level *K*_1_ *within-subject* %CV, for each ROI as a function of iteration, for IR and DR methods for the lower count levels: 20% (*n* = 5), 10% (*n* = 10), and 5% (*n* = 10). %CV calculated using corresponding ROI mean at iteration 2 of IR at full counts. The *y*-axis is on a log-scale and its range varies across plots.

**Figure 6.**
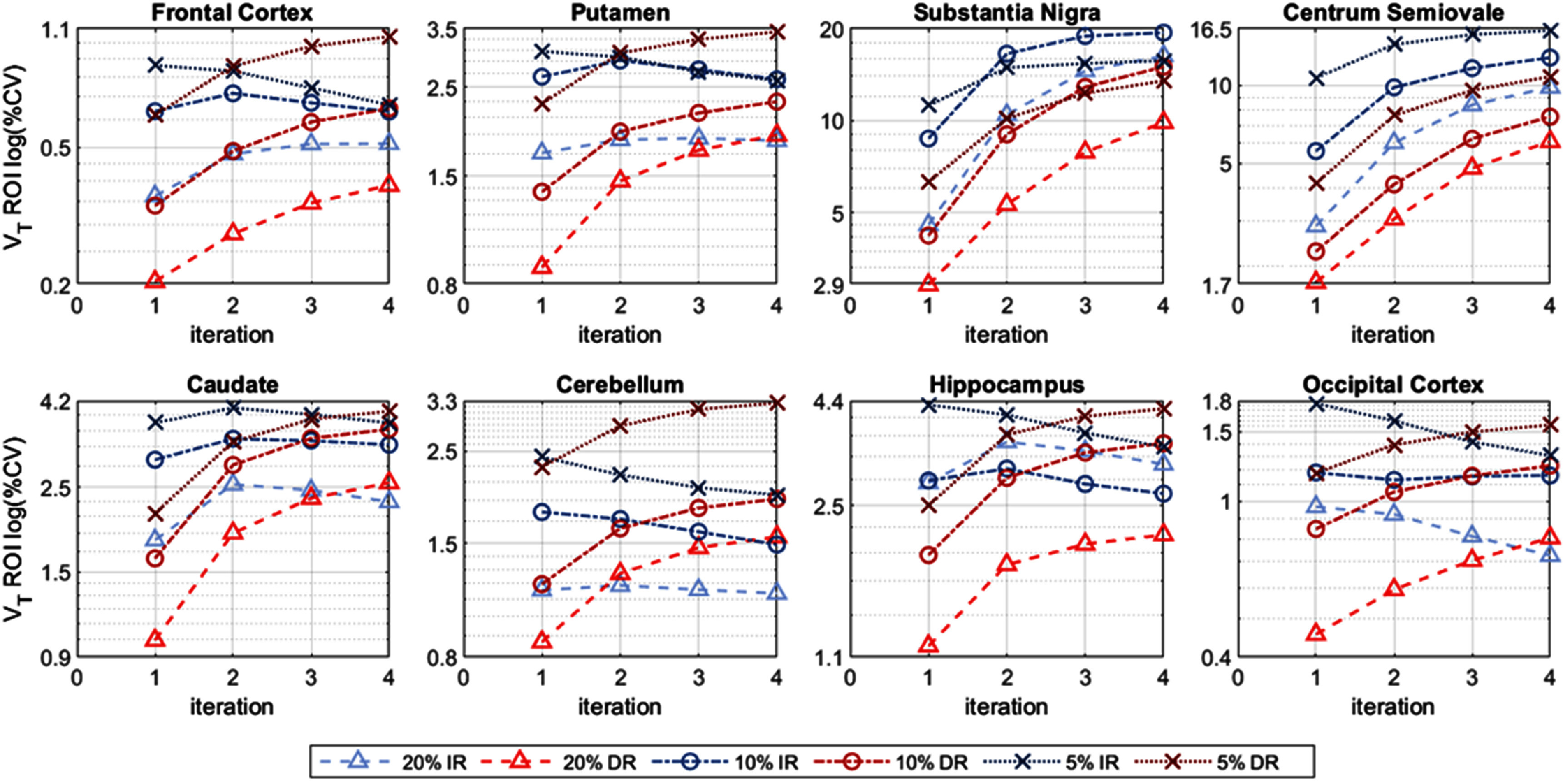
ROI-level *V*_T_ *within-subject* %CV, for each ROI as a function of iteration, for IR and DR methods for the lower count levels: 20% (*n* = 5), 10% (*n* = 10), and 5% (*n* = 10). %CV calculated using corresponding ROI mean at iteration 2 of IR at full counts. The *y*-axis is on a log-scale and its range varies across plots.

The *within-subject* %CV, at the ROI level, is shown in figure 5 (*K*_1_), figure S12 (*k*_2_) and figure 6 (*V*_T_), for the 8 ROIs, as a function of iteration, for each count level. Obviously, the ROI-level %CV values are lower than their voxel-wise counterparts and the %CV values are lower with larger ROIs.

Whilst *K*_1_ %CV values are similar between IR and DR at iteration 2 for the 20% count level, as the iteration increases and count level decreases, the IR variability increases. DR delivered relatively more stable *K*_1_ %CV values across count levels and iterations. Similar observations were made for *k*_2_ although the noise advantage of DR over IR was slightly greater than for *K*_1_ (figure S12).

Interestingly, the pattern for the *within-subject* %CV for *V*_T_ at the ROI level (figure 6) is more variable, especially for IR. The *V*_T_ %CV values for IR unexpectedly decreased at higher iterations for the frontal cortex, putamen, caudate, cerebellum, hippocampus, and occipital cortex (figure 6). Furthermore, the %CV for the hippocampus was slightly lower at 10% counts compared to 20% counts for the IR, while the %CV for the substantia nigra was lower at 5% counts compared to 10% counts for IR, and also for DR at iteration 4 (figure 6). Finally, the %CV for DR is higher than IR at 5% counts for the cerebellum. We attribute these counter-intuitive observations to the constrained range of *k*_2_ values used in the basis function implementation for creation of parametric images (see discussion).

### 3.3. Between-subject variability

#### 3.3.1. Voxel-wise between-subject variability

**Figure 7.**
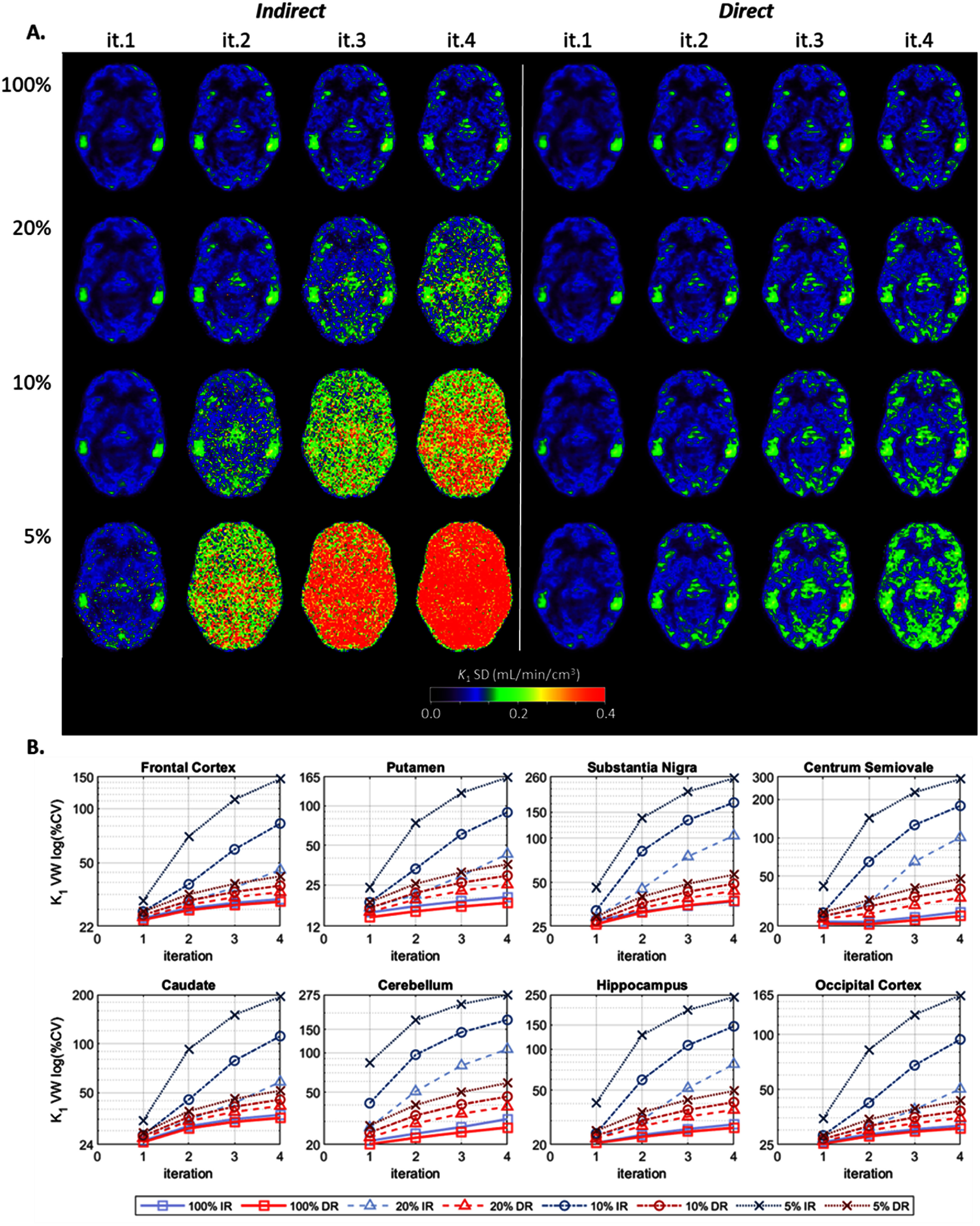
(A) *K*_1_ *between-subject* SD images, calculated across eight subjects for each noisy replicate (equations (6) and (7)) and then averaged across replicates per count level (equations (8) and (9)), for IR (left) and DR(right) methods, for iterations 1–4, and for each count level: 100% (*n* = 1), 20% (*n* = 5), 10% (*n* = 10), and 5% (*n* = 10). (B) Voxel-wise (VW) percent coefficient of variation (%CV), calculated using corresponding ROI mean at iteration 2 of IR at full counts, for each ROI as a function of iteration. The *y*-axis is on a log-scale and its range varies across plots. Clearly the variability is lower for DR across all subjects.

Figure 7(A) displays the *between-subject* SD images, for each noisy replicate (equations (6) and (7)) and then averaged across replicates (equations (8) and (9)), for *K*_1_ per count level and each iteration. Figure 7(B) shows the corresponding voxel-wise (VW) %CV, for the 8 ROIs.

At the full count level, the DR and IR images are visually comparable. IR delivered slightly higher *between-subject* variability with an increase in %CV (IR vs DR) of 4% ± 3% at iteration 2 and 6% ± 5% at iteration 4. For the lower count levels, the *between-subject K*_1_ variability is clearly higher for IR than DR especially with count levels decreasing and iterations increasing. At iteration 2, the average percent increase in variability of IR over DR was 20% ± 25% for the 20% count level and 221% ± 90% for the 5% count level. At iteration 4, this percent increase was 102 ± 64% and 346 ± 82% for the 20% and 5% counts respectively. As observed for the within-subject case above, the %CV delivered by DR at 5% counts, was comparable to, or even lower than, that of IR at 20% counts.

Similar behavior was observed for *k*_2_, but with higher %CV values (see supplement figure S13).

**Figure 8.**
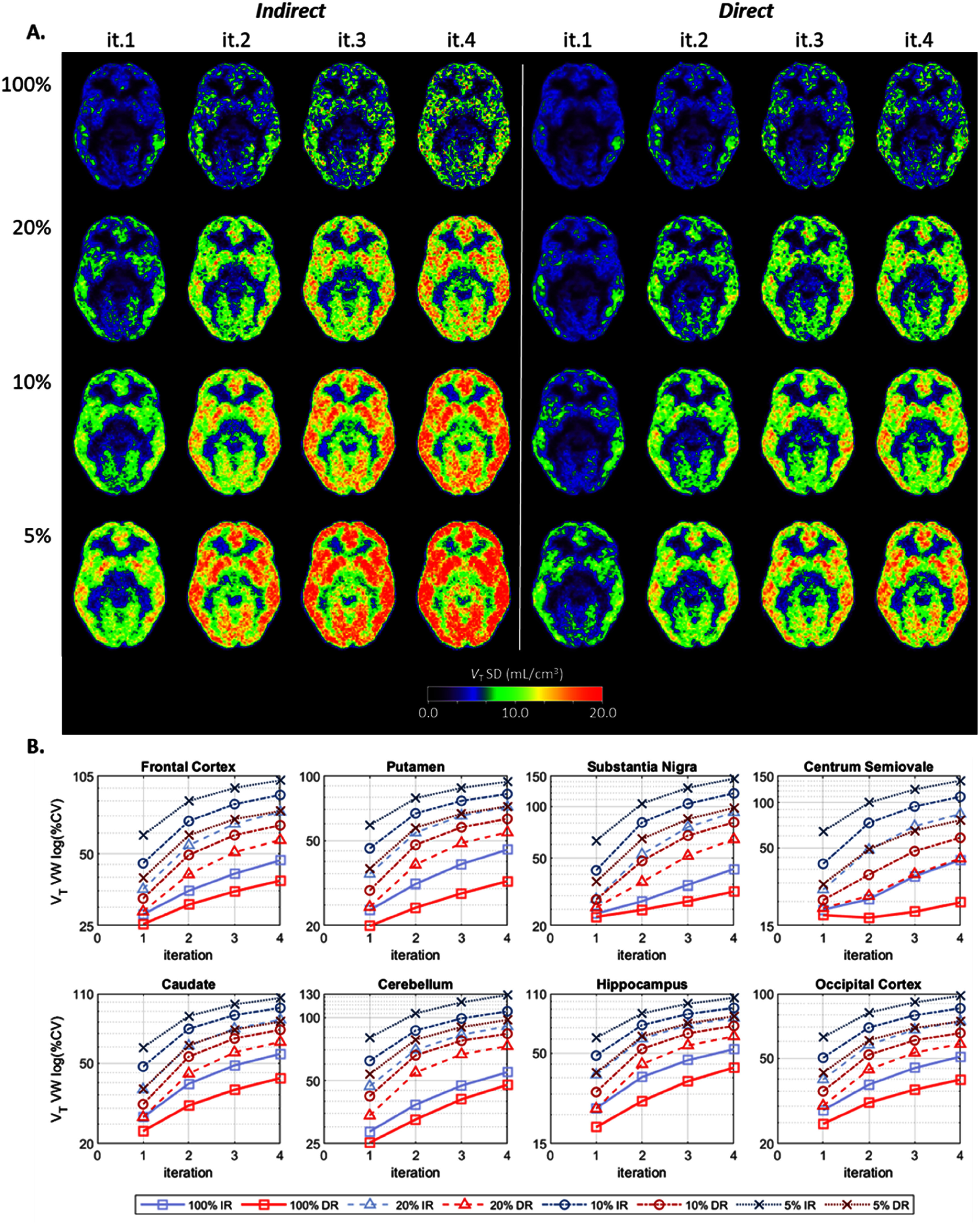
(A) *V*_T_ *between-subject* SD images, calculated across eight subjects for each noisy replicate (equations (6) and (7)) and then averaged across replicates per count level (equations (8) and (9)), for IR (left) and DR (right) methods, for iterations 1–4, and for each count level: 100% (*n* = 1), 20% (*n* = 5), 10% (*n* = 10), and 5% (*n* = 10). (B) Voxel-wise (VW) percent coefficient of variation (%CV), calculated using corresponding ROI mean at iteration 2 of IR at full counts, for each ROI as a function of iteration. The *y*-axis is on a log-scale and its range varies across plots. Clearly the variability is lower for DR across all subjects.

Figure 8(A) displays the *between-subject* SD parametric images for *V*_T_ per count level and each iteration. Figure 8(B) shows the corresponding voxel-wise (VW) %CV, for the 8 ROIs, as a function of iteration, for each count level. IR clearly delivered higher *between-subject* variability compared to DR even at the full count level.

At full counts, the percent increase in variability delivered by IR (compared to DR) was 24% ± 9% at iteration 2 and 36% ± 24% at iteration 4 across all 8 ROIs. For the lower count levels, at iteration 2, this increase was 45% ± 24% for the 20% count level and 48% ± 24% for the 5% count level. At iteration 4, the increase was 39% ± 25% and 40% ± 19% for the 20% and 5% count levels respectively. As observed for the within-subject case above, the %CV delivered by DR at 5% counts was also comparable to that of IR at 20% counts.

#### 3.3.2. ROI-level between-subject variability

**Figure 9.**
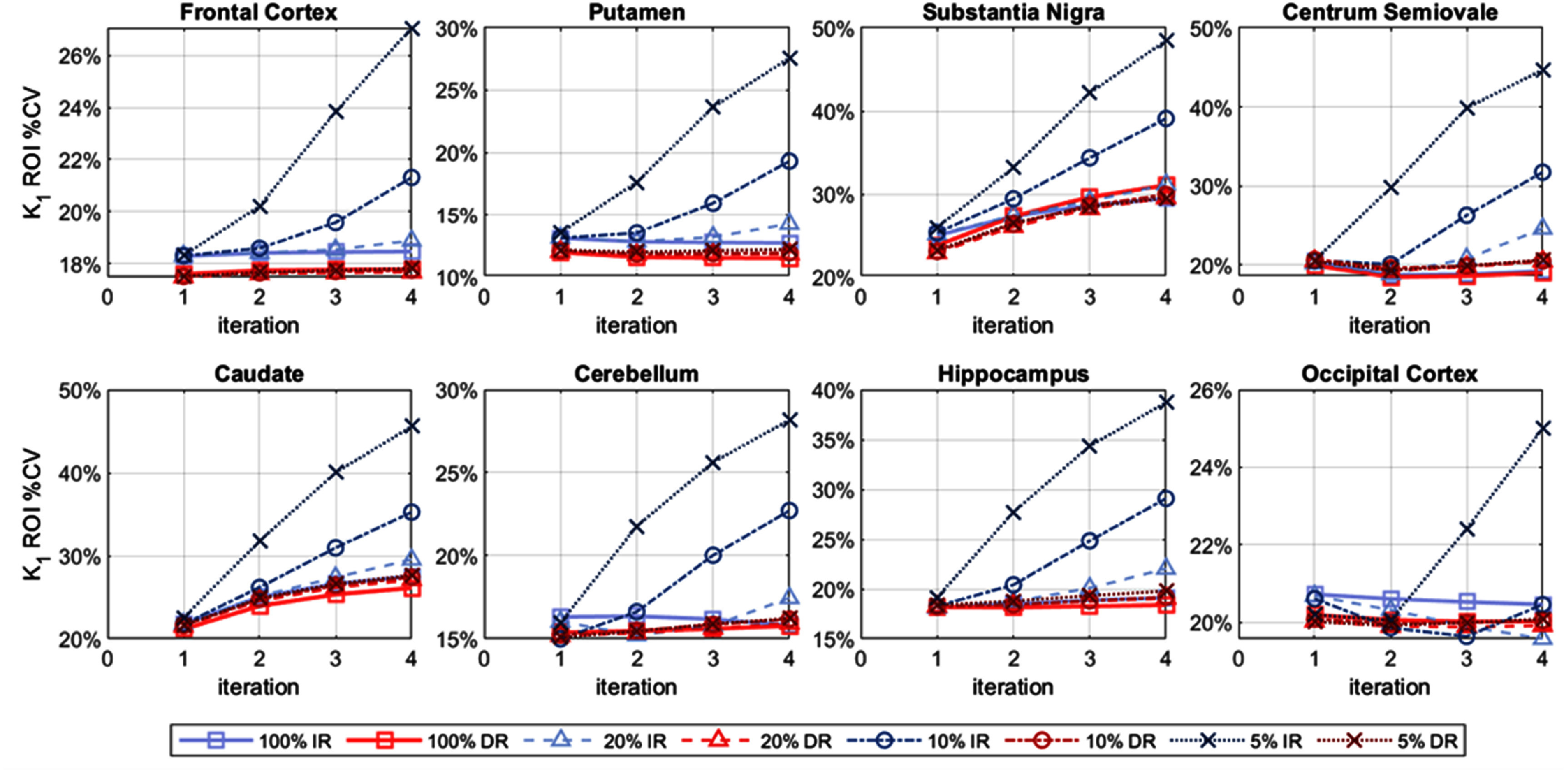
ROI-level *K*_1_ *between-subject* %CV, for each ROI as a function of iteration, for IR and DR methods for each count level: 100% (*n* = 1), 20% (*n* = 5), 10% (*n* = 10), and 5% (*n* = 10). %CV calculated using corresponding ROI mean at iteration 2 of IR at full counts. The *y*-axis range varies across plots.

**Figure 10.**
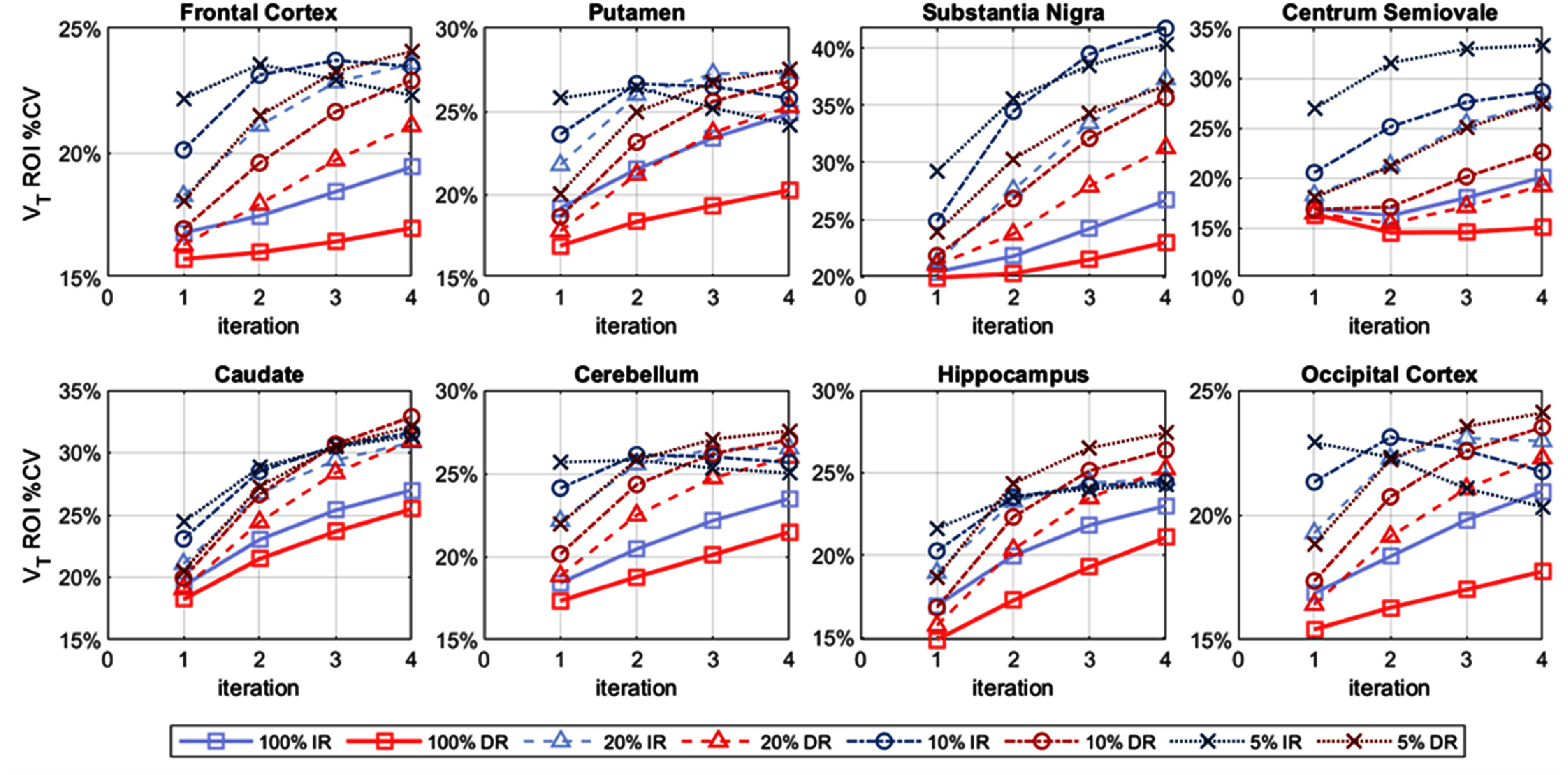
ROI-level *V*_T_ *between-subject* %CV, for each ROI as a function of iteration, for IR and DR methods for each count level: 100% (*n* = 1), 20% (*n* = 5), 10% (*n* = 10), and 5% (*n* = 10). %CV calculated using corresponding ROI mean at iteration 2 of IR at full counts. The *y*-axis range varies across plots.

The *between-subject* %CV at the ROI level is shown in figures 9 (*K*_1_), S14 (*k*_2_) and 10 (*V*_T_), as a function of iteration, for each count level. Note that the ROI-level *between-subject* variability is considerably higher than the corresponding *within-subject*, due to the presence of biological variability across subjects. Thus, the advantage of DR over IR is less dramatic than that shown by within-subject %CV analysis. DR delivered relatively more stable, and generally lower, *K*_1_ and *k*_2_ %CV values, across count levels and iterations. The pattern for the *between-subject* %CV for *V*_T_ at the ROI level (figure 10) is more variable, especially for IR, as observed for the within-subject case (figure 6). Interestingly, the *between-subject V*_T_ %CV values for IR decreased at higher iterations, e.g. for the frontal and occipital cortices, and in some cases, the %CV for the lower count data was less than that of the higher count data. We again attribute these observations to the constrained range of *k*_2_ used in the basis function implementation (see discussion).

## 4. Discussion

In this work, we evaluated direct-4D reconstruction (DR) compared to the conventional indirect-3D (IR) method with a group of human datasets for the 1TC model using downsampling of the list mode data. The performance of DR compared with IR on kinetic parameter estimates, was evaluated for both within-subject (across noisy realizations) and between-subject variability, as well as on the average values, at the voxel and ROI levels. Overall, DR offers considerable improvement over IR by reducing the magnitude of noise in the reconstruction, and also dramatically reducing noise-induced bias for *K*_1_ and *V*_T_ parameters, both for *within-subject* and *between-subject* analyses. To our knowledge, this is the first study to compare the performance of DR with IR, not only for within-subject variability but also for between-subject, as well as assessing the performance at the ROI level as opposed to voxel-wise level only.

### 4.1. Average values

Even when considering average ROI or voxel values, DR outperformed IR by a reduction in bias for both *K*_1_ and *k*_2_ (figures 1 and S1). The simulation allowed us to explore noise-induced bias with an estimation algorithm identical in structure to that used in IR. However, the present simulation used Gaussian-distributed data and cannot be used to quantitatively predict the magnitude of bias in reconstructed PET images derived from Poisson-distributed raw count data. For *V*_T_, there is a noise-induced bias for both IR and DR, but less so for DR (figure 2). Since *V*_T_ is calculated as the ratio of *K*_1_ and *k*_2_, a bias is unavoidable as noise in the denominator increases. The simulation delivered smaller *K*_1_ and *k*_2_ bias than the real data set (figures S5 and S6). We attribute this primarily to the difference in the statistical distribution of the simulated and real data sets. Figure S15 shows the histograms of the voxel values for one subject (putamen at iteration 2 and frame 13), for all realizations per count levels, compared to the corresponding values used in the simulation. As the noise increased, the real data delivered more skewed distributions, i.e. becoming non-normally distributed, whereas the simulated data were normally distributed. Nevertheless, the simulation confirmed the presence of noise-induced bias. This noise-induced bias observed primarily with IR suggests that between-subject variation in noise levels due to injected dose and body size can introduce subject-specific bias.

### 4.2. Voxel-wise variability

At the voxel level, DR clearly delivered lower variability compared to IR, and that for both the within-subject (figures 3, 4 and S8) and between-subject analyses (figures 7, 8 and S13), and for all three kinetic parameters. Furthermore, the variability delivered by DR at the 5% count level, is equivalent to, or even lower, than that of IR at 20% counts, for all iterations. In part, this is due to the optimal handling of the Poisson statistics in the list mode data by DR In addition, IR using weighted least squares suffers from the non-Gaussian nature of the low-count reconstructed data (figure S15) with the additional challenge of proper selection of data weights. This demonstrates the advantage of DR not just for the within-subject case as commonly reported, but also at the between-subject level.

### 4.3. ROI-level variability

The ROI level assessment was performed by extracting ROI values from parametric images. Two interesting observations were noted:

(1) DR generally delivered comparable or lower variability for *K*_1_ and *k*_2_ compared to IR at the ROI level for both the within-subject (figures 5 and S12) and between-subject analyses (figures 9 and S14). However, this was not the case for *V*_T_ where IR unexpectedly delivered lower %CV for certain ROIs, especially at the 10% and 5% count levels (figures 6 and 10), even when the parametric SD *V*_T_ images clearly show lower variability for DR than IR (figures 4 and 8).
(2) Whilst the %CV increased with iteration and noise level for *K*_1_ and *k*_2_, the variability for IR *V*_T_ decreased at higher iterations for the low count data set (figures 6 and 10).

We attribute this counterintuitive behavior to the constrained, but biologically justified (Finnema *et al* 2018), basis function range (*k*_2_ = [0.005–1.0] min^−1^) used to create the parametric images using the 1TC model (Lodge *et al* 2000, Yan *et al* 2012, Germino *et al* 2017). With more iterations, noise increases, causing the kinetic estimation for the IR method (because it does not model the noise properly) to hit the lower boundary of the *k*_2_ basis function range. This therefore delivers voxels with the same biased *k*_2_ values, leading to larger bias in *V*_T_ and simultaneously produces artificially lower variability for IR.

**Figure 11.**
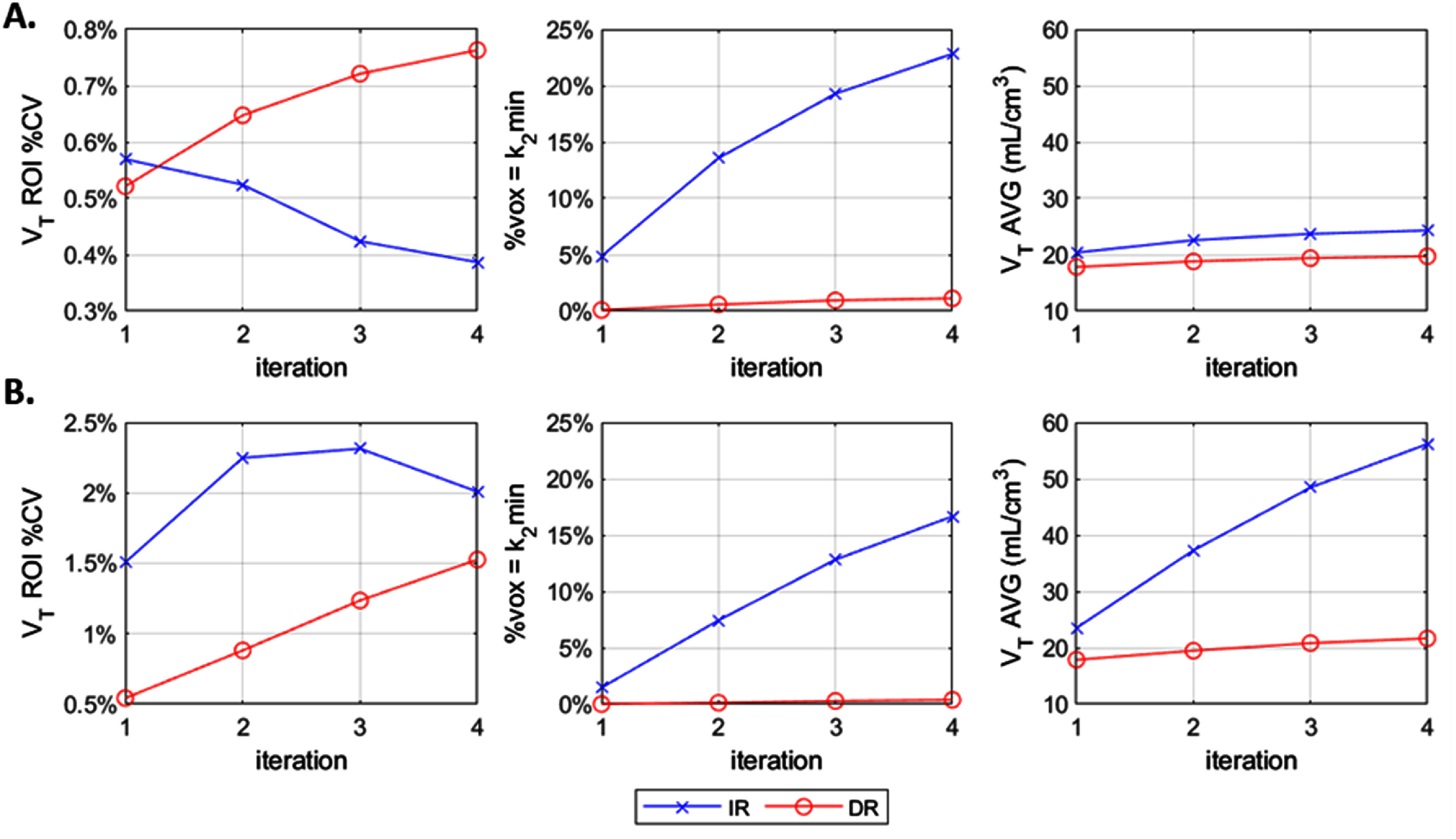
ROI-level *V*_T_ %CV (left), percent of voxels in the ROI hitting the lower boundary of the *k*_2_ basis function range (middle) and average *V*_T_ values (right), for a single-subject, calculated across 10 noisy replicates (10% count level), for the frontal cortex as a function of iteration, for IR and DR reconstruction methods. (A). Default *k*_2_ minimum vof 0.005 min^−1^. (B). Lowered *k*_2_ minimum to 0.001 min^−1^.%CV calculated using ROI mean at iteration 2 of IR at full counts.

To support these observations, we focused on the 10% count level for the frontal cortex in one subject, and compared the ROI-level *V*_T_ metrics using the default basis function range (*k*_2_ = [0.005–1.0] min^−1^), and then with an extended range by decreasing the lower boundary to *k*_2_ = 0.001 min^−1^. Figure 11 shows the results with ROI-level *V*_T_ %CV values (left), the percent of voxels hitting the lower *k*_2_ boundary of the basis functions (middle), and the average *V*_T_ values (right). The results for the default basis function range are shown in panel (A) and for the lower minimum *k*_2_ shown in panel (B). In panel A, the ROI-level *V*_T_ %CV (across iterations) for IR is unexpectedly lower than that of DR, and is decreasing with higher iterations. On the other hand, when the lower boundary is decreased (panel B), IR *V*_T_ %CV becomes higher than that of DR, and increases with iterations. It can be observed that the number of voxels hitting the lower boundary (middle graphs) decreases when lowering the minimum *k*_2_ value. So, it would be reasonable to ask why was not the lower *k*_2_ boundary used as default. This choice is explained by the right graphs of *V*_T_ average, where the bias introduced by calculating *V*_T_ as *K*_1_/*k*_2_ with small, noisy *k*_2_ values produces unrealistically highly biased *V*_T_ values. Conversely, the metrics between panels A and B for DR are only slightly impacted, which again demonstrates the advantage of DR over IR both in terms of reduced variability and bias.

### 4.4. Limitations and future directions

#### 4.4.1. Number of iterations

In this work, we did not go beyond 4 iterations (using 30 subsets) for both IR and DR as justified by the per-iteration changes in *K*_1_ and *k*_2_ values being less than 2% (IR and DR), and in *V*_T_ being about 4% (IR) and 3% (DR), after 3 iterations at the full count level (tables S1–S3).

Furthermore, in practice, we do not consider having more iterations to be useful, especially at low counts. For example, for IR, the *V*_T_ voxel noise level at 10% counts exceeded 50% by 2 iterations (figure 8), which also introduces biased ROI values due to the *k*_2_ cut-off used in the kinetic modeling.

#### 4.4.2. Scatter estimation

Scatter estimation and corresponding scatter scale factors were updated iteratively at subsets 0, 4, and 10 of the first iteration. Consequently, for DR, scatter correction relies on emission images generated from the current, non-converged *K*_1_ and *k*_2_ estimates, which could potentially introduce bias in the scatter calculation, propagating to following iterations and introducing errors in the final parametric maps. Nevertheless, if the DR scatter correction implementation was introducing a bias compared to IR, we would have expected to see a notable difference in mean ROI values between DR and IR for the full count data. However, these between-method differences were small: at iteration 2, 3% ± 2% for *K*_1_ and −5% ± 3% for *V*_T_. Nonetheless, a more extensive evaluation of scatter estimation for both IR and DR would be useful to assess the impact on the final parametric images.

#### 4.4.3. Randoms fraction for downsampled data

When downsampling full count list mode data, the randoms fraction cannot be reduced. Thus, our low-count replicates were in fact conservative, in that the randoms fraction was higher than what would be found in real low-count scans. Nevertheless, we do not believe this affects our results, since the same downsampled datasets were used for both IR and DR

#### 4.4.4. Resolution modeling: spatially invariant PSF

The current PSF implementation for the HRRT in MOLAR is spatially invariant, as has been applied for all data acquired on the HRRT at Yale. The same spatially invariant PSF was used for both IR and DR methods, so we expect PSF related effects to be similar between IR and DR nevertheless, a spatially variant model has indeed been shown to deliver qualitative improvements for the reconstructed images, in terms of resolution recovery and noise suppression, however small increases in ringing artefacts were observed (Angelis *et al* 2015). Furthermore, the PSFs should ideally be isotope-specific for accurate resolution modeling (Kotasidis *et al* 2014). Therefore, even if the spatially invariant model may be less optimal, it was shown to be sufficient for the HRRT (Sureau *et al* 2008).

#### 4.4.5. Between-group comparison

The next step for this work is to investigate the impact of DR vs IR on a between-group comparison between PD patients and healthy controls (HCs). Using the substantia nigra as an example, a small region that has previously been shown to be involved in PD (Matuskey *et al* 2020, Holmes *et al* 2024), we performed a preliminary power analysis to assess group differences in *V*_T_ as a function of dose and reconstruction method. The number of subjects required for a power of 80% (*α* = 0.05) was 8 for DR, as opposed to 11 for IR, at full counts iteration 2, using a mean *V*_T_ for HCs of 8.72 ml cm^−3^ (Matuskey *et al* 2020), and for PD of 6.41 ml cm^−3^ (IR) or 6.21 ml cm^−3^ (DR) (figure 2). For that calculation, the group SD for HC was set to the voxel-level between-subject SD for PD of 1.79 ml cm^−3^ (IR) or 1.60 ml cm^−3^ (DR) (figure 8). At 10% counts, the number of subjects becomes 25 for DR, but 80 for IR, assuming the same mean *V*_T_ values but using the 10% count SD *V*_T_ values for PD of 5.17 ml cm^−3^ (IR) or 3.08 ml cm^−3^ (DR). At full counts, the variability is dominated by the differences between subjects, while at 10% counts it becomes dominated by the noise. This emphasizes the benefits of DR on noisy data.

#### 4.4.6. DR with machine learning

Machine learning methods have demonstrated substantial improvements in parametric image quality (Li *et al* 2023, Liu *et al* 2024, Hong *et al* 2025, Muller *et al* 2025). Development of such methods for either IR or DR requires a well-trained network that reduces noise, which may impact the Poisson nature of the data, without introducing bias and degrading spatial resolution. We believe that a key challenge will be proper handling of varying noise levels. The intrinsic noise level in the data varies by scan based on injected dose and subject body size, and varies over the scan time, especially for C-11 tracers. If the machine learning methods focus on noise reduction, they may produce more resolution loss when count levels are low, yielding time-dependent and subject-dependent variations in resolution. Time-dependent resolution variation can introduce bias in the kinetic fitting. Nevertheless, if done properly, we expect that the combination of DR with machine learning methods will provide even larger advantages over conventional IR.

#### 4.4.7. Further dose reduction

Finally, it would be interesting to assess the impact of our DR method on data acquired on the NeuroEXPLORER, a next-generation human brain-dedicated PET imager with high-sensitivity (Li *et al* 2024, Omidvari *et al* 2025), in order to see how much lower we could downsample the listmode data, and thus the injected dose, without introducing significant bias.

## 5. Conclusion

In this work we have demonstrated the benefits of direct-4D reconstruction, over conventional indirect-3D reconstruction, which delivered considerably lower variance. At 5% counts, the variability delivered by DR was equivalent to, or even lower than, that of IR at 20% counts. To the best of our knowledge, this is the first demonstration that direct-4D reconstruction can deliver lower variability for between-subject analysis as well as within-subject analysis. In addition, bias was much more substantial for IR than DR, in part due to noise-induced bias in the kinetic parameter estimation, as confirmed by a simulation study.

The benefits of DR could in principle allow for a lower injected dose while still delivering unbiased results. This would allow for more scanning sessions, when necessary, such as multiple conditions or multiple radiotracers performed in certain studies, while still being below the safe radiation exposure limit. Furthermore, with studies in pediatric populations being of interest, decreasing the dose while keeping result integrity is of key importance. Finally, we expect to have increased statistical power with DR compared to IR, so studies can be performed with fewer subjects.

With these advantages and potential benefits, additional research and application of DR for dynamic parametric imaging is warranted.

## Data Availability

The data cannot be made publicly available upon publication because they are owned by a third party and the terms of use prevent public distribution. The data that support the findings of this study are available upon reasonable request from the authors. Suppl.Mat. Direct-4D PET in PD available at https://doi.org/10.1088/1361-6560/ae520a/data1.
